# Supplementary material for: Comprehensive Transcriptome Analysis of Responses during Cold Stress in Wheat (Triticum aestivum L.)
Source: Genes (Basel). 2023 Mar 31;14(4):844. doi: 10.3390/genes14040844 (PMC10137996; doi:10.3390/genes14040844)
Supplement: Supplementary file 1 [file genes-14-00844-s001.zip › Figure S1.pdf]

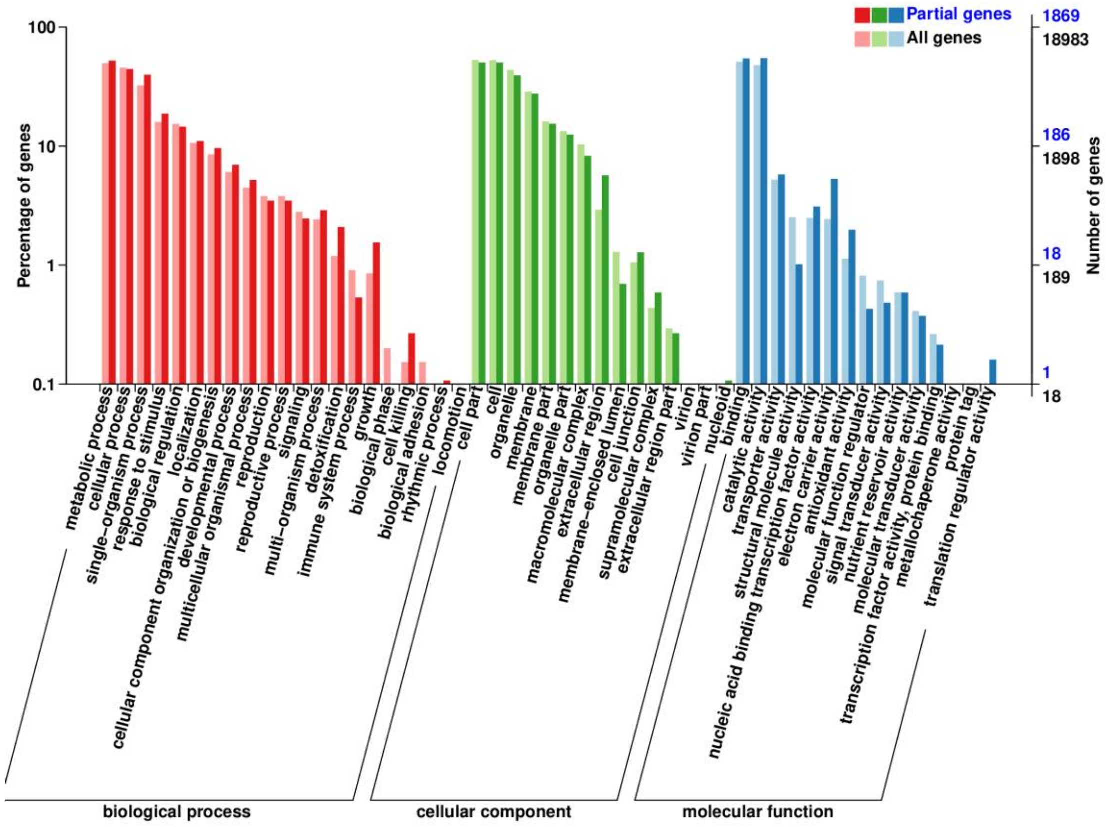

Figure S1. Functional classification of the differentially expressed genes (DEGs) referring to the Gene Ontology (GO) database
