# Supplementary material for: Comprehensive Transcriptome Analysis of Responses during Cold Stress in Wheat (Triticum aestivum L.)
Source: Genes (Basel). 2023 Mar 31;14(4):844. doi: 10.3390/genes14040844 (PMC10137996; doi:10.3390/genes14040844)
Supplement: Supplementary file 1 [file genes-14-00844-s001.zip › Figure S2.pdf]

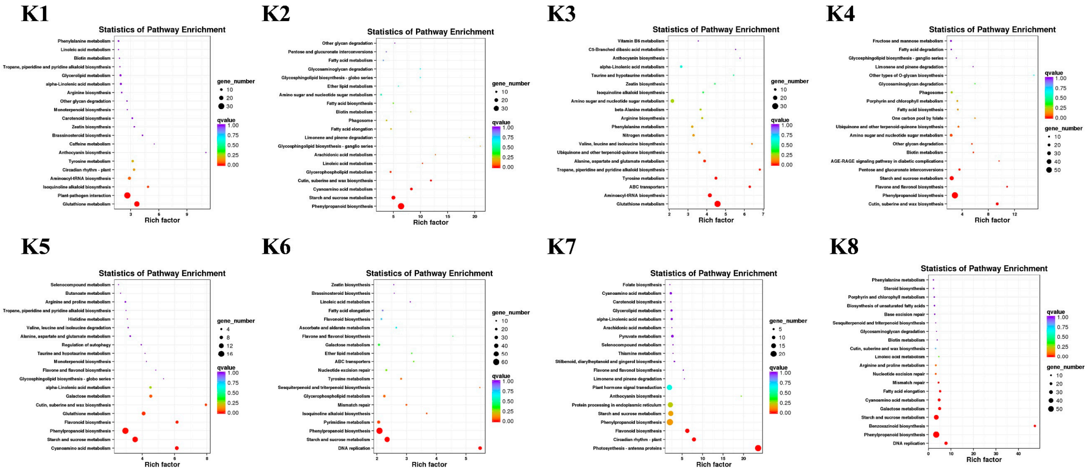


**Figure S2.** Differentially expressed genes (DEGs) involved top 20 enriched pathways of the eight *K*-means clusters referring to KEGG database
