# Supplementary material for: Comprehensive Transcriptome Analysis of Responses during Cold Stress in Wheat (Triticum aestivum L.)
Source: Genes (Basel). 2023 Mar 31;14(4):844. doi: 10.3390/genes14040844 (PMC10137996; doi:10.3390/genes14040844)
Supplement: Supplementary file 1 [file genes-14-00844-s001.zip › Figure S3.pdf]

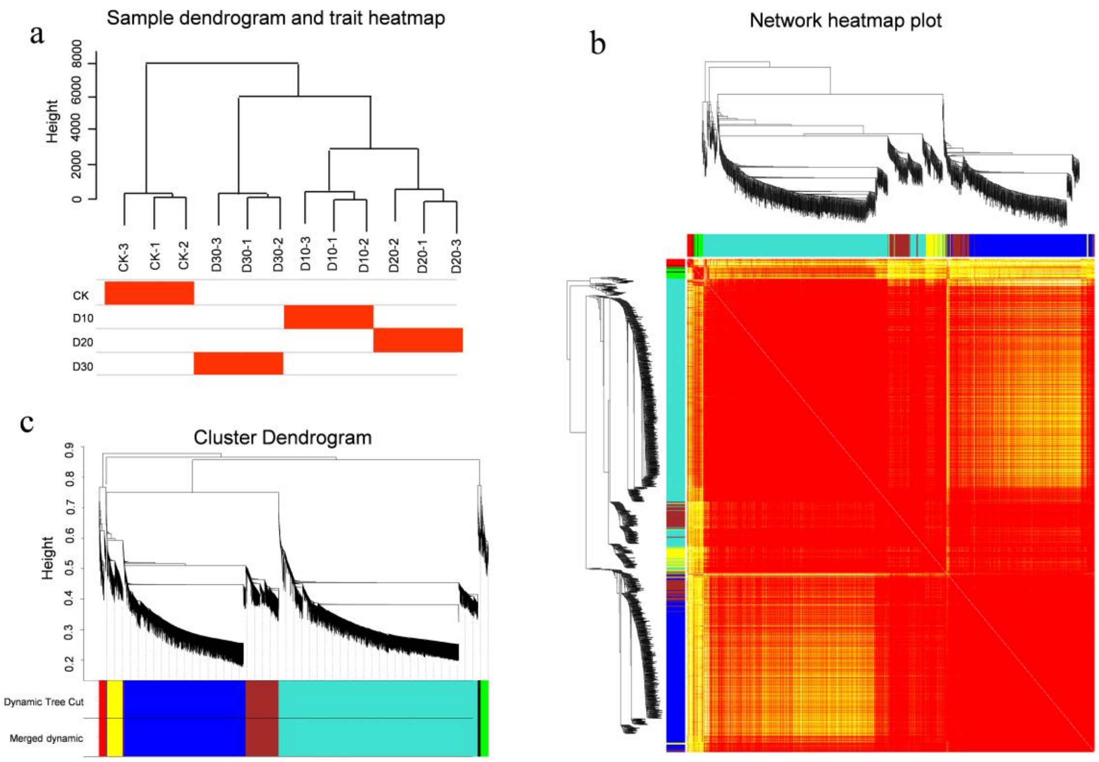

Figure S3. Screening the key genes of cold treatment changes with WGCNA

(a)The sample clustering and correlation coefficients revealed strong repeatability among the biological replicates, and no outlier needed to be removed

(b)the visualization of modules by color scheme

(c) co-expression modules of associated with target traits
